# Is owning your home good for your health? Evidence from exogenous variations in subsidies in England

**DOI:** 10.1016/j.ehb.2020.100903

**Published:** 2020-12

**Authors:** Luke A. Munford, Eleonora Fichera, Matt Sutton

**Affiliations:** a School of Health Sciences, University of Manchester, UK; b Department of Economics, University of Bath, UK

**Keywords:** Home ownership, Health and well-being, Right to Buy, Housing policies

## Abstract

Home ownership is an important component of wealth and may affect health through a range of mechanisms. Using macro- and micro-level data from 2000 to 2008, we seek to estimate the causal effect of home ownership on health by exploiting the Right to Buy policy, which encouraged long-term tenants of publicly rented housing to buy their home at a large discount. At the macro-level we find that a 10 percentage-point increase in home ownership rates is associated with a 2 percentage-point reduction in the number of people reporting having a longstanding health condition. At the individual level we find that home ownership increases the General Health Questionnaire score by 1.46 points on a 37-point scale and self-assessed health by 0.19 points on a 5-point scale and reduces the number of health conditions reported by 0.65. Further analyses show that home ownership affects health via labour markets, with new job opportunities, extra time saved travelling and resources available for healthy leisure activities. These results suggest that housing policies, such as affordable housing, can be an example of non-health policies that improve health.

## Highlights

• The decision to own your home can affect your health and well-being.
• Little is known about this causal relationship or possible explanations.
• We provide evidence at area and individual level that home ownership is goods for health.
• Possible explanations are better labour market outcomes and healthier lifestyles.

## 1 Introduction

Home ownership is a sizable component of wealth in Western economies, but is it also important for health? Housing wealth represents 60 % of the financial wealth of households in Britain (Banks et al., 2003), and equity extraction from unsold homes reached 6–8 % of total household income in the mid-2000s (Reinold, 2011). Such wealth gains have an ambiguous effect on health depending on the relative size of the substitution and wealth effects. Fichera and Gathergood (2016) find that house price gains improve physical health. However, home ownership could also have detrimental effects on health if home owners become anxious about keeping up with mortgage payments (Nettleton and Burrows, 2000; Evans et al., 2003).

Another potential channel through which home ownership could impact on health is via labour markets (Blanchflower and Oswald, 2013; Laamanen, 2017; Oswald, 1996). Blanchflower and Oswald (2013) found that a doubling of the home ownership rate was associated with more than a doubling of the long-run unemployment rate. Longer commuting times, which may be a result of home ownership, have also been found to reduce well-being and reduce health-related quality of life (Munford et al., 2015).

Nonetheless, home ownership could improve health through better housing conditions. There is evidence of a detrimental effect of poor housing conditions (e.g. temperature and humidity) on respiratory health (Marmot et al., 2008; Shaw, 2004). As home owners can make structural adjustments to their dwellings, home ownership could improve housing quality and, therefore, health (Haurin et al., 2002; Chapman, 2013). Home ownership could also impact psychological health through social comparisons, by providing people with a sense of physical and emotional security, control over their life and safety (Elsinga et al., 2008), and social capital through increased participation in church and community organisations (Homenuck, 1973), neighbourhood and block associations and socialisation (Rohe and Stegman, 1994; Fischer, 1982), and political activity (Glaeser and Sacerdote, 2000).

Whilst there is some evidence that housing is associated with health (Parliamentary Office of Science and Technology, 2011; Buck et al., 2016; Marmot et al., 2010), there is little causal evidence about the effect of home ownership on health (Dietz and Haurin, 2003). Establishing a causal relation between health and home ownership is difficult because healthier individuals might select into home ownership and because home ownership rates might simply capture area-level factors, such as unemployment rates and possibly other omitted factors, affecting both health and ownership.

We exploit variations in government house purchase subsidies in England under a policy called ‘Right to Buy’. These discounts, representing more than 76 % of an average yearly salary, constituted a substantial incentive to buy a home. This policy increased home ownership as a share of housing tenure by 15 percentage points and generated the largest contribution to privatisation revenue in the UK. It represents an exceptional home-ownership intervention in international terms, though the Israeli government introduced similar reforms in 2000 (Hausman et al., 2020). Nonetheless, it has not been analysed much by economists, except for two studies examining its effects on the quality and quantity of publicly-provided houses and on mobility using the British Household Panel Survey (Disney and Luo, 2017; van Ham et al., 2013).

We examine the effect of home ownership on health with macro and micro level panel data using either Local Authorities or individuals as our observational units. We find that home ownership improves physical and psychological health. We explore potential mechanisms and find that the health effects of home ownership operate through labour markets, with home owners more likely to become employed and spend less time travelling to work. Home owners also spend more money on leisure and are less likely to smoke and suffer from lifestyle-related diseases.

## 2 Literature review

Our paper is closely related to two existing themes of literature: (1) the ways in which housing more generally affects health and (2) the effects of exogenous variations in wealth on health. We summarise each in turn below.

### 2.1 Channels through which home ownership could affect health

How does home ownership affect health? In economic models, health is produced by human capital investments, lifestyle behaviours and other random shocks, and it can be influenced by socioeconomic factors such as income and wealth (Grossman, 1972). Assume that individuals maximise a utility function with health and consumption of other goods subject to time and budget constraints. In this case, individuals will allocate time and resources for health investments to equalize the marginal utility to the marginal cost. In such a model, wealth gains behave like permanent income shocks shifting the budget constraint out and affecting health.

However, there are at least five reasons why the direction of this relation is ambiguous. First, there is a direct effect of home ownership on health through housing conditions. Disney and Luo (2017) showed that the Right to Buy lowered housing quality for residual public renters who did not partake in the scheme. There is evidence of a detrimental effect of poor housing conditions on health (Marmot et al., 2008; Shaw, 2004). Shaw (2004) provides a review of potential direct and indirect effects of housing on health looking at historical and current evidence. She points out that respiratory health is the main health outcome to be affected by temperature and humidity in the house. Palacios et al. (2020) have recently shown that living in a poorly-maintained dwelling is associated with poorer self-assessed health and more doctor visits, particularly amongst older people. As home owners are able to make structural adjustments to their dwellings, home ownership could improve housing quality and, therefore, health (Haurin et al., 2002; Chapman, 2013).

Second, home ownership could also have a direct effect on health by providing people with a sense of physical and emotional security, control over their life and safety (Elsinga et al., 2008). This might reflect social comparisons with those who did not make it onto the housing ladder or it might be related to home owners' ability to make changes to their houses. These non-financial effects may affect psychological health favourably.

Third, home ownership could have an indirect effect on health through a housing wealth effect. Housing wealth represents 60 % of British households' financial wealth (Banks et al., 2003). The U.K. housing market is one of the most volatile in the world (Ferrari and Rae, 2011). Equity extraction from unsold homes is quite large in the U.K., reaching 6–8 % of total household income in the mid-2000s (Reinold, 2011). Such wealth gains have an ambiguous effect on health depending on the relative size of the substitution and wealth effects. Fichera and Gathergood (2016) find that house price gains improve physical health. They find no statistically significant effect of housing wealth gains on risky health behaviours such as smoking and drinking. But they find that housing wealth increases the likelihood of private medical coverage for home owners. However, this wealth effect could have detrimental effects on health inducing anxiety if home owners struggle to keep up with mortgage payments (Nettleton and Burrows, 2000; Evans et al., 2003).

Another potential indirect effect of home ownership on health is via labour markets (Blanchflower and Oswald, 2013; Laamanen, 2017; Oswald, 1996). Blanchflower and Oswald (2013) find that the housing market can create dampening externalities on the labour market and the economy. Using historic state-level data in the United States, they show that states with higher rates of home ownership have longer commute times and higher levels of joblessness. There is indeed evidence that longer commuting times reduce well-being, have detrimental effects on self-assessed health and reduce health-related quality of life (Munford et al., 2015). The effect found by Blanchflower and Oswald (2013) is quite large, as a doubling of the home ownership rate is associated with more than a doubling of the long-run unemployment rate. These results are confirmed by micro-level data on two million individuals from the March Current Population Surveys (1992–2011). Laamanen (2017) find similar results using Finnish individual level data exploiting a rental housing market deregulation reform in the early 1990s. However, when looking at the effect of house price gains in the U.K., Fichera and Gathergood (2016) find a reduction of working hours by women suggesting a substitution of working hours with the additional wealth.

A final indirect effect of home ownership is via the production of social capital. Some studies find that home owners are more likely to belong to church and community organisations (Homenuck, 1973); they are involved in neighbourhood and block associations (Rohe and Stegman, 1994); they are more socially communicative with neighbours (Fischer, 1982) and politically active (Glaeser and Sacerdote, 2000). This social activity effect could favourably impact psychological health.

### 2.2 Effects of other exogenous variations in wealth on health

Our paper also relates to a more general literature, the relationship between economic resources and health. One strand of this literature uses exogenous changes in economic resources exploiting lottery wins (Apouey and Clark, 2015; Lindhal, 2005; Gardner and Oswald, 2007), inheritance (Meer et al., 2003; Kim and Ruhm, 2012), cohort-level income shocks (Adda et al., 2009), weather shocks (Fichera and Savage, 2015), spousal wealth (Michaud and Van Soest, 2008) and recessions (Ruhm, 2000).

Apouey and Clark (2015) use a sample of lottery winners from the British Household Panel Survey (BHPS) between 1997 and 2005 and find that greater lottery winnings produce better mental health, but induce riskier lifestyle choices such as smoking and social drinking. Meer et al. (2003) use the 1984, 1989, 1994 and 1999 waves of the Panel Study of Income Dynamics and an instrumental variable approach where inheritance is an instrument for wealth. They find that in the short-run there is no statistically significant evidence of the health-wealth nexus. The seminal work of Ruhm (2000) suggests that unemployment rates and health are pro-cyclical, but later work (Ruhm, 2015) showed that this was true only after accounting for time periods, with different effects in the periods 1976–1995 compared to 1991−2010. It has also been argued that the level of analysis (micro vs. macro) is important, but van den Berg et al. (2017) find consistent results when they use the same data at both levels.

A second strand of this literature exploits changes in public policies as source of exogenous variation in income or wealth (Snyder and Evans, 2006; Frijters et al., 2005; Case, 2004; Schmeiser, 2009). For instance, Frijters et al. (2005) compare health satisfaction between East and West Germany using post-unification income changes. Using data from the German Socio-Economic Panel Survey between 1984 and 2002, they find positive effects of income changes on health satisfaction. Schmeiser (2009) exploits state-level differences in the Earned Income Tax Credit supplement to examine the impact of income on body mass index (BMI). Using the U.S. National Longitudinal Survey of Youth 1979 cohort and instrumental variable methods, he finds that an additional $1000 of family income raises BMI by 0.07 units for men and by 0.24 units for women.

Finally, our paper contributes to a growing number of studies that exploits the timing and spatial variations of a policy (Hoynes and Schanzenbach, 2009; Ludwig and Miller, 2007; Cascio et al., 2010; Fichera and von Hinke, 2020). For instance, Fichera and von Hinke (2020) use the variation in the introduction of food labelling across British supermarkets to examine its impact on household dietary choices. In our paper we exploit the timing and variation in the intensity of the discount across geographical areas in England.

## 3 The Right to Buy policy

The Right to Buy policy gives long-term tenants of publicly-owned properties the legal right to buy their residence at a large discount. The rationale was to give households a tangible asset, secure their finances and improve public finances as well.

Eligibility for the scheme depends on the length of time that individuals have rented their property. No discounts are available if the property is rented privately. The size of discount available is related to the property value, the property type and the length of tenancy, and is subject to a maximum cap.

Over the period that we study, eligibility required at least two years of tenancy. For houses, the possible discount was calculated as 32 % of the property value, plus 1% for each year of tenancy over two years. For flats, the possible discount was 44 % of the property value plus 2% for each year of tenancy over two years.

**Fig. 1:**
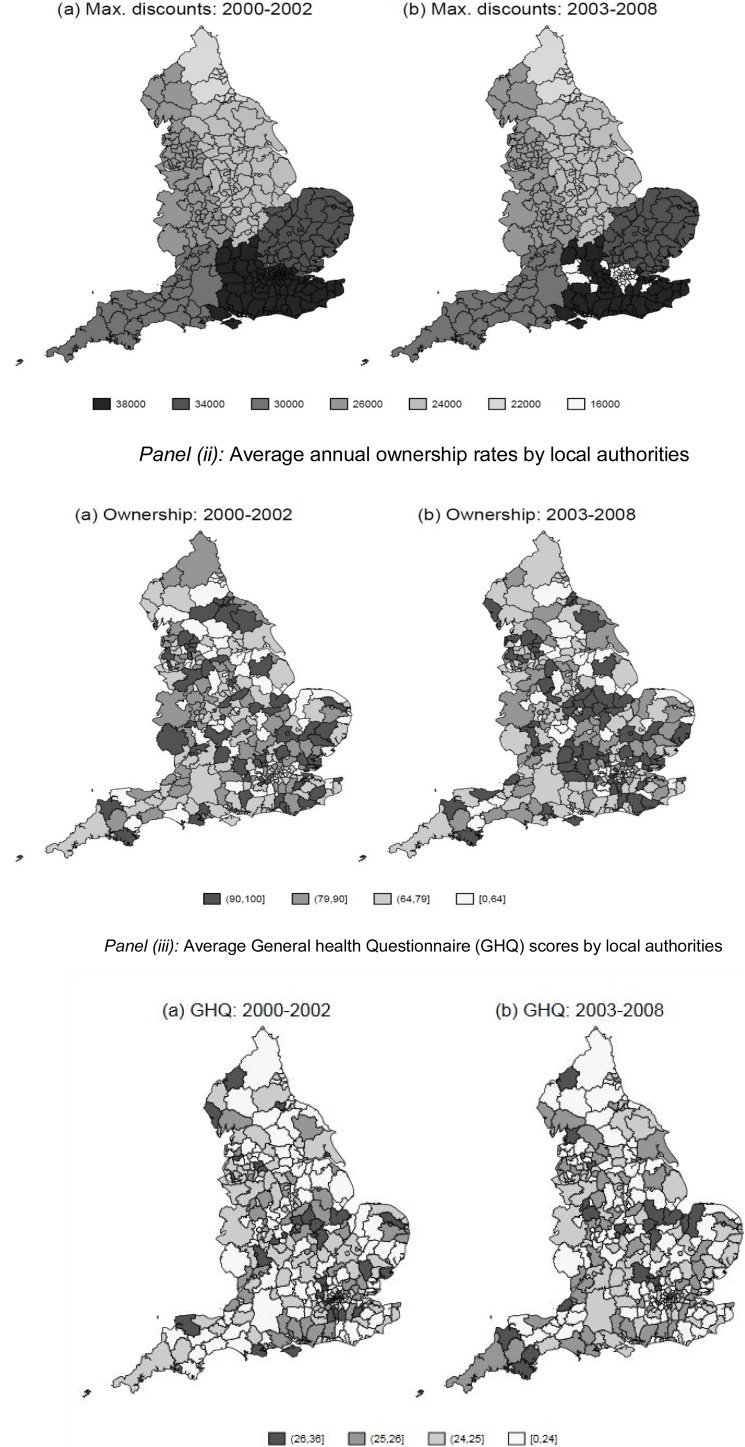
Map of geographic variation in subsidies and ownership rates in England. *Panel (i):* Right to Buy maximum discount caps by local authorities *Panel (ii):* Average annual ownership rates by local authorities *Panel (iii):* Average General health Questionnaire (GHQ) scores by local authorities

Source: Authors' representation. Data for the two graphs in panel (i) has been obtained from the Department of Communities and Local Government. The two graphs in panel (ii) use BHPS data 1999–2008, to show the local authority average of the ownership variable. As this variable is binary, we multiply by 100 to obtain percentages. The two graphs in panel (iii) use BHPS data 1999–2008, to show the local authority average of the GHQ variable. This variable is increasing in health, with 36 the highest possible response.

Between February 1999 and February 2003, the maximum discount was capped at £38,000 in most areas, though this cap varied geographically and was as low as £22,000 in some areas. In March 2003, the discounts were reduced to reflect pressure on available public housing in some areas. In nine Local Authorities (LAs) in the South East, and all but two London boroughs, the maximum discount was reduced to £16,000 (see Fig. 1, Panel (i)).

To illustrate how the scheme works, consider two identical individuals, A and B, living in houses valued at £100,000 in 2001. Both have been public renters in their respective homes for 7 years. Without caps, they would both be entitled to a Right to Buy discount of 0.32*(£100,000)+0.01*(7−2)*(£100,000) = £37,000. Individual A lives in the south east and so can have the full £37,000 discount, as this is just below the cap of £38,000. However, as individual B lives in the north-east, their discount is capped at £22,000.

In Panel (ii) of Fig. 1 we show that the local authority homeownership rates are higher in areas where the discount were higher (correlation = 0.05). This is particularly true within the East of England and the South East regions. However, when the discounts were reduced in 2003, the correlation with ownership rates fell as well (correlation = 0.03).

The size of the maximum discount in each local authority was set nationally by central government. There is evidence of geographical clustering of the cap (see Fig. 1 panel (i)), as they were set according to average property values and measures of economic prosperity. To check whether these caps were functions of health in the local area, we regressed the caps on measures of population health, economic indicators and year dummies. The health measures (contemporaneous or lagged) were not significant predictors of the discount in any specification (Table A2, Appendix A (in Supplementary material)). We therefore treat these maximum discount caps as conditionally exogenous to health.

## 4 Datasets

### 4.1 Local authority data

We start by considering the Local Authority District (LAD) as the unit of analysis. These 326 areas are administrative geographies that usually encompass one city or a larger rural area.

We use annual data from 2000 to 2010 on home ownership, including: (i) the proportion of individuals who own their home (either outright or through a mortgage); (ii) the number of recorded Right to Buy sales; and (iii) the maximum available Right to Buy discount cap.

We also obtain two measures of population health: (i) the proportion of individuals who report having a longstanding health condition; and (ii) the average number of health conditions reported on a pre-specified list.

We add other characteristics which could correlate with health, including: (i) the proportion of people who are economically active; (ii) median hours worked per-week; (iii) median weekly pay; (iv) population size; and (v) the proportion of the population aged 65 years and older.

Further information on these variables, and their sources, are reported in Table A1 (in Supplementary material).

### 4.2 Individual level data: The British Household Panel Survey

At the individual level, we use data from waves 10–18 (2000–2008) of the British Household Panel Survey (BHPS). We did not use the follow-up years in Understanding Society (the UK Household Longitudinal Study) because the Great Recession, characterised by a subprime mortgage crisis, had a differential effect across the UK potentially confounding our analysis. The BHPS is a nationally-representative, annual, longitudinal survey of households in the UK. Each member (aged 16+) of the household is asked a series of questions on a wide range of topics. Information is also collected at the household level (including household size and composition, council tax band.^1^

One advantage of the BHPS is that it asks respondents about a broad range of health conditions and contains detailed information on housing, geographic location and a broad range of socio-economic characteristics, such as income and labour market status. It also has the attraction of being a panel survey which employs a ‘following rule’, so that it remains representative of the UK population throughout the 18 waves. In the following subsections we outline some of the more relevant variables we consider. Additional detail on further variables considered is included in Appendix A.2 (in Supplementary material).

We use a special license version of the data containing the LAD location of each household. Using this information, we match in data on house price sales. We use the LAD-level house price index provided by Halifax Bank of Scotland (Fichera and Gathergood (2016) for details).

#### 4.2.1 Health and well-being outcomes

We use self-assessed health (SAH) as a measure of subjective health (Contoyannis et al., 2004). Individuals are asked “*Please think back over the last 12 months about how your health has been. Compared to people of your own age, would you say that your health has on the whole been …*”, and are given options (1) ‘*Excellent*’ through to (5) ‘*Very Poor’*. For ease of interpretation, we recode SAH such that higher scores correspond to better health.

We use the General Health Questionnaire (GHQ) to measure subjective well-being. The GHQ contains 12 questions designed to identify minor psychiatric disorders and measure psychosocial health and has been widely used as a proxy for well-being (Clark and Oswald, 1994; Clark, 2003; Roberts et al., 2011). Each of the 12 questions is answered on a 0–3 scale, thus giving a 37-point summary scale. As with SAH, we reverse code this, so that higher responses correspond to higher levels of health.

We use the count of the number of reported health conditions as a more objective measure of health. We focus on the 13 conditions that were consistently presented to respondents throughout the survey period. In additional analyses, we investigate each of these conditions separately by generating five dummy variables for the following categories: (a) musculoskeletal problems, comprising arthritic/rheumatic conditions; (b) cardiovascular diseases (CVD), comprising diabetes and heart/blood pressure problems; (c) skin, allergy, hearing and sight problems; (d) respiratory problems, comprising bronchial and asthmatic conditions; and (e) other chronic problems, comprising cancer, stroke and epilepsy.

We measure health-seeking behaviours with five variables: (a) possession of supplementary private health insurance, (b) number of General Practitioner visits, (c) current smoking status, (d) number of cigarettes smoked per day, and (e) whether physically active. In the BHPS individuals are asked how many times they visited the doctor in the last 12 months with the possible answers being: none; one or two times; three to five times; six to ten times; and more than ten times. We recode this variable to the midpoint value of each interval of reported number of visits. Active is a dummy variable that equals one if in the past 12 months the BHPS has been gardening or she had done yoga or sport several times a year or more.

#### 4.2.2 Housing tenure, housing characteristics, and eligibility for Right to Buy

The BHPS asks people to report their housing tenure from a seven-point list. We use this information to classify people into three groups: (1) owners (including outright and with a mortgage); (2) public renters; and (3) private renters.

If a house is owned, the owner is asked to report the value of their property and the band within which it falls for the payment of local council tax. We generate dummy variables for each of the eight council tax bands: “A” ≤£40,000, “B” >£40,000 and ≤£52,000, “C” >£52,000 and ≤£68,000, “D” >£68,000 and ≤£88,000, “E” >£88,000 and ≤£120,000, “F” >£120,000 and ≤£160,000, “G” >£160,000 and ≤£320,000, and “H” >£320,000. Non-owners are not asked to report the value of the property, but are asked to report the council tax band, as this is payable regardless of tenure type.

As well as recording the date of the interview, the BHPS asks individuals what date they moved into their current house. From these two pieces of information, we can calculate how long an individual has lived at their current address. We use this information to establish which public renters are eligible for the Right to Buy discount, and the size of discount they are eligible for.

Characteristics of the house are reported, including number of rooms, property type (detached, semi-detached, terrace, end-terrace purpose built flat, converted flat, contains business premises, and other), whether there is central heating (and if so, what fuel), whether there is a garden or terrace (“*Does this accommodation have the following facilities? A place to seat outside e.g. a terrace or garden*?”), if there is a separate kitchen, and if there is a separate toilet. Individuals are also asked to report if there is a problem with pollution and crime/vandalism in their local area.

#### 4.2.3 Estimation sample at the individual level

As we are interested in the transition from renting to owning, our estimation sample is comprised of people who were public renters when we first observe them in the data. We only include individuals with more than one observation. Our estimation sample contains 1204 individuals and 6430 observations, around 12 % of the whole BHPS sample.

## 5 Empirical strategy

### 5.1 Area-level analysis

Our starting point is an area-level analysis. The basic model is:

(1) $$H_{lt}={\text{α}}_{1}owner_{lt}+{\text{α}}_{2}{\text{X}}_{\text{l}\text{t}}+{\text{μ}}_{l}+{\text{u}}_{\text{t}}+{\text{ε}}_{\text{l}\text{t}}$$

where *H* is a measure of health for area *l* at time *t*, *owner* is the home ownership rate, *X* is a vector of time varying characteristics (see Table A1 (in Supplementary material)), μ is a LAD fixed-effect, *u* is a time fixed-effect, and ε is an error term.

However, the above simple specification ignores possible reverse causality between health and home ownership. To overcome this, we implement a fixed-effects instrumental variable (FE-IV) specification, where the first stage is specified as:

(2) $$\text{o}\text{w}\text{n}\text{e}{\text{r}}_{\text{l}\text{t}}={\text{π}}_{1}RtBDiscountCap+{\text{π}}_{2}{\text{X}}_{\text{l}\text{t}}+{\text{μ}}_{\text{l}}^{1}+{\text{u}}_{\text{t}}^{1}+{\text{ε}}_{\text{l}\text{t}}^{1}$$

where the first-stage instrument, *RtBDiscountCap*, is the maximum discount. The identifying assumption is that the RtB discount should only affect health through its effect in encouraging home ownership.

### 5.2 Individual-level analysis

At the individual level, we exploit the longitudinal nature of the dataset and control for a wide range of factors that might affect both home ownership and health:

(3) $$H_{ilt}={\text{β}}_{1}\text{o}\text{w}\text{n}\text{e}{\text{r}}_{\text{i}\text{l}\text{t}}+{\text{β}}_{2}{\text{X}}_{\text{i}\text{l}\text{t}}+{\text{β}}_{3}\text{H}{\text{C}}_{\text{i}\text{l}\text{t}}+{\text{μ}}_{\left(\text{l}\times \text{t}\right)}+{\text{ε}}_{\text{i}\text{l}\text{t}}$$

where subscript *i* indicates the individual, *l* is the region where *i* lives, and *t* is year. *H* is a measure of health or well-being, *owner* is a binary variable equal to one if an individual owns the house they live in. The vector *X* contains socioeconomic and demographic information known to correlate with health and well-being (Appendix A.2 (in Supplementary material)). *HC* contains selected house characteristics that might have a direct effect on health (central heating fuel type, if there is a garden, if there are issues with pollution or if there are issues with crime/vandalism). $\mu_{\left(l\times t\right)}$ is the interaction of region and time fixed effects and $\varepsilon_{ilt}$ is a stochastic error term.

The coefficient $\beta_{1}$ is our main coefficient of interest indicating the relationship between home ownership and health. By exploiting the longitudinal nature of our data, we estimate within region changes in health rather than health differences between regions. As we cannot control for all the geographic factors that correlate both with home ownership and health, we follow Lovenheim and Mumford (2013) and include region-by-time fixed effects. These allow us to control for factors such as the quality of healthcare or of schooling which might change over time and affect health and the propensity to become a home owner.

However, $\beta_{1}$ is still likely to be biased as unobserved factors that affect health also affect home ownership. For instance, individuals’ time preferences influence how individuals make intertemporal choices such as investing in a house, becoming a home owner and investing in prevention to increase life expectancy.

To overcome these issues, we modify Eq. (3) as follows:

(4) $$H_{ilt}=\beta_{1}owner_{ilt}+\beta_{2}X_{ilt}+\beta_{3}HC_{ilt}+{\text{β}}_{4}{\hat{\text{φ}}}_{\text{i}\text{l}\text{t}}+\mu_{\left(l\times t\right)}+\varepsilon_{ilt}$$

where the predicted residual (${\hat{\varphi }}_{ilt}$) is obtained from the following hedonic regression:

(5) $$owner_{ilt}={\text{γ}}_{1}\text{R}\text{t}\text{B}\text{D}\text{i}\text{s}\text{c}\text{o}\text{u}\text{n}{\text{t}}_{\text{i}\text{l}\text{t}}+{\text{γ}}_{2}{\text{C}}_{\text{i}\text{l}\text{t}}+{\text{γ}}_{3}X_{ilt}+e_{ilt}$$

The probability of becoming a home owner is explained by the potential Right to Buy discount that this individual could receive if they bought the property they currently publicly rent. As stated above, this discount varies by individual (and house), by LAD, and over time. We describe the hedonic regressions associated with this discount in the next subsection. The vector *C* contains other factors that could influence the choice to buy a house, including the duration at the current property and the average local area house price.

The identifying assumption is that, after controlling for a range of individual and house characteristics, as well as time invariant and time varying local authority factors, home ownership is conditionally exogenous to health. Under this assumption, $\beta_{1}$ in Eq. (4) measures the effect of home ownership on health through changes in the value of the discount they would be entitled to (Terza et al., 2008).

Note that Eq. (5) is a pooled regression and we do not use individual level fixed-effects. This is because there were too few individuals whose ownership status changed. However, given the estimation sample comprises of people who were initially public renters, any change in the ‘owner’ dummy is from an initial position of renting – i.e. owner = 1 implies a person is now (in period t) an owner, given they were previously a renter.

#### 5.2.1 Calculating the size of the potential Right to Buy discount

As the Right to Buy discount varies across both time and place, we first calculate the potential discount renters could be entitled to. To do this, we use several hedonic regressions.

##### 5.2.1.1 Step 1: calculate the estimated value of a rented property

Property values are only reported by owners. We therefore need to estimate property values for renters. We regress the house prices (*HP*) reported by owners on house characteristics (HC*) and the local house prices (from the land registry; $\bar{HP}$).

(6) $$HP_{ilt}={\text{δ}}_{1}\text{H}{\text{C}}_{\text{i}\text{l}\text{t}}^{\text{*}}+{\text{δ}}_{2}{\bar{HP}}_{ilt}+u_{ilt}$$

where $u_{ilt}$ is a stochastic error term. The elements of the vector HC* include: the number of rooms interacted with the house type, the council tax band, the central heating fuel type, if there is a separate toilet/bathroom, if the kitchen is open-plan, if there is a garden/terrace, if there is an indoor toilet, and if there are neighbourhood problems with either crime/vandalism and/or pollution/the environment. We do not include time or local authority fixed-effects, as this variation should be captured in the local house price (${\bar{HP}}_{ilt}$).

We then apply the estimated coefficients ($\hat{{\text{δ}}_{1}},\hat{{\text{δ}}_{2}}$) to the same house characteristics of renters, to obtain an imputed value of a rented property, ($\hat{\text{H}\text{P}}$).

##### 5.2.1.2 Step 2: calculate the potential Right to Buy discount

Once we have the estimated value of a rented property ($\hat{\text{H}\text{P}}$), we can use this to calculate the potential Right to Buy discount that this individual could receive if they bought this property. These discounts are:

(7) $${\text{R}\text{t}\text{B}\text{D}\text{i}\text{s}\text{c}\text{o}\text{u}\text{n}\text{t}}_{\text{i}\text{l}\text{t}}\;=\;\left\{\begin{array}{c} min\{M_{lt},\;((0.32+0.01(\text{max}\left(T_{ilt}-2\right),0)\hat{)HP_{ilt}}\}\\ min\{M_{lt},\;((0.44+0.02\left(\text{m}\text{a}\text{x}(T_{ilt}-2\right),0))\hat{HP_{ilt}}\} \end{array}\right)\;\begin{array}{c} if\;a\;house\\ if\;a\;flat\; \end{array}$$

Where *M* is the maximum discount cap in local authority *l* at time *t*, and *T* is the length of time that the individual has lived in the current property.

### 5.3 Robustness checks

To mitigate concerns about other within-area factors that may affect health, we estimate Eq. (4) for private renters, a “placebo” group that we expect not to be affected by the Right to Buy. We use propensity score matching with caliper and no replacement to construct a sample of private renters similar to public renters in terms of age, marital status, education, income and household size, house characteristics and region and time interactions.

A second potential source of concern relates to the measurement error of house characteristics being related to health. Therefore, we regress reported house price on average local house prices (Eq. (6)) excluding the house characteristics.

In a third robustness check we exclude income as it may be a “bad control” (Angrist and Pischke, 2008).

Fourth, we investigate the health effects of home ownership separately for heads and non-heads of household.

Fifth, one might argue that the Right to Buy scheme was more appealing to people who are in employment as opposed to those who are not. We re-estimate equation Eq. (4) on those who were employed the first time they were observed in the survey.

Sixth, to account for the potential ordinal nature of SAH we apply an ordered logit model in the second stage.

Finally, we perform other robustness checks by removing outliers such as Manchester and Birmingham, West Devon and South Buckinghamshire from our macro-level analyses. We also check the robustness of our results to leads and lags, as we do not know if there were anticipation effects, nor do we know if our results are due to increasing health of new owners or deteriorating health of non-owners.

## 6 Results

### 6.1 Descriptive statistics

Descriptive statistics for the area level dataset are provided in Table 1, panel (a). The average ownership rate is just over 70 % and the average number of self-reported conditions is 0.58. Just under 30 % of people report having a longstanding health condition.

**Table 1:** Summary statistics from the LAD and individual-level data.

	Estimation Sample					Full Sample
Panel (a): LAD-level	Mean	S.D.	Min.		Max.	
Rate of people with a longstanding health condition	0.28	0.05	0.00		0.58	
Average number of self-reported health conditions	0.58	0.18	0.00		2.16	
Home ownership rate	0.71	0.12	0.08		0.96	
RtB discount cap	32,967	7026	16,000		38,000	
Prop. of population aged 65+ years	0.17	0.04	0.06		0.30	
Median weekly pay (deflated)	388.29	73.23	222.50		884.04	
Median weekly hours worked	39.60	0.81	29.4		40	
Economic activity rate (aged 16−64)	0.78	0.05	0.59		0.93	
Prop. of population with no qualifications	0.12	0.05	0.02		0.32	
Crimes per 1000 population	92.55	4.61	42.35		1377.0	
LADs (N)	311					
Observations (N*T)	2161					
Panel (b): Individual-level	Mean	S.D.	Min.		Max.	Mean (S.D.)
*Health and well-being*						
Self-assessed health *SAH)	3.45	1.02	1		5	3.81 (0.92)
GHQ	23.74	6.04	0		36	24.82 (5.38)
Number of Health Conditions	1.69	1.59	0		9	1.18 (1.32)
*Individual characteristics*						
Male	0.42		0		1	0.47
Age	50.45	19.93	16		99	47.41 (18.27)
Age squared	2942.1	2145.52	256		9801	2581.73 (1880.20)
Married	0.42		0		1	0.57
School qualifications	0.33		0		1	0.32
College qualifications	0.10		0		1	0.19
University qualifications	0.05		0		1	0.22
*House characteristics*						
Equivalised log household income	6.8	0.47	1.58		9.76	7.11 (0.62)
Number of people in household	2.68	1.5	1		8	2.69 (1.30)
Estimated House Price (£'000 s)	120.05	78.5	0.23		717.1	N/A
Calculated RtB discount (£'000 s)	24.25	9.89	0		38	N/A
LAD average house prices (£'000 s)	142.98	73.51	43.27		823.18	N/A
*Transition to ownership*						
Becomes a home owner	0.17	0.35	0		1	N/A

Panel (c): Mechanisms	Mean		S.D.	Min.	Max.	Mean (S.D.)
Private Health Insurance	0.02			0	1	0.06
Number of visits to the doctor in last year	3.82		3.52	0	10	2.93 (3.06)
Number of cigarettes smoked per day	16.16		8.99	0	80	14.30 (8.39)
Current smoker	0.43			0	1	0.24
Active	0.36			0	1	0.37
Employed	0.43			0	1	0.62
Working time (hours per week)	33.78		12.74	1	97	34.64 (12.23)
Commuting time	20.57		18.55	1	240	24.86 (22.02)
Expenditure on leisure (£ per week)	28.39		36.72	0	160	41.30 (43.18)
Housing costs (net monthly £)	188.89		184.69	0	1716	266.97 (315.98)
Garden	0.91			0	1	0.94
Number of rooms	3.67		1.20	1	10	3.66
Type of accommodation: detached	0.02			0	1	0.02
Type of accommodation: Semi-detached	0.33			0	1	0.33
Type of accommodation: End-Terrace	0.11			0	1	0.12
Type of accommodation: Terrace	0.29			0	1	0.28
Type of accommodation: Purpose built flat	0.22			0	1	0.22
Type of accommodation: Converted flat	0.03			0	1	0.03
Council Tax band: A	0.51			0	1	0.53
Council Tax band: B	0.24			0	1	0.23
Council Tax band: C	0.13			0	1	0.13
Council Tax band: D	0.08			0	1	0.08
Council Tax band: E	0.02			0	1	0.02
Council Tax band: F	0.01			0	1	0.01
Council Tax band: G	0.002			0	1	0.002
Council Tax band: H	0.001			0	1	0.001
Central heating type: gas	0.84			0	1	0.84
Central heating type: electricity	0.12			0	1	0.12
Central heating type: solid fuel	0.02			0	1	0.02
Central heating type: oil	0.01			0	1	0.01
Central heating type: other	0.06			0	1	0.001
Own bathroom	0.98			0	1	0.98
Separate kitchen	0.99			0	1	0.99
Individual toilet	0.99			0	1	0.99
Pollution in area	0.08			0	1	0.07
Vandalism in area	0.27			0	1	0.16
Vote	0.35			0	1	0.39
Talk to neighbours	0.81			0	1	0.78
Satisfaction with home	5.13		1.62	1	7	5.40 (1.38)
Individuals (N)	1204					20,294
Observations (N*T)	6430					56,607

These values are based on the original sample(s), and not on the bootstrapped data. In panel (b), for the full sample, we report mean and standard deviation values only for reasons of space. In Panel (c) the sample size for each variable is the same as the estimation samples in Table 6 which we do not report for reasons of space. The full sample for the mechanisms is lower than the one reported for the labour market variables referring to those in work (NT = 34,700), and for the cigarettes smoked by smokers (NT = 12,986).

Of the 1204 individuals in our micro-level sample, 207 (17 %) go on to become home owners (Table 1, panel (b)). Of the 207 people who become owners, only 25 (12 %) then go back to renting at some point in the future.

The average calculated house value in our sample is just over £120,000 and the predicted Right to Buy discount is £24,250. Rosen and Rosen (1980) modelled owned and rented houses as two distinct commodities, with different characteristics (i.e. size, outside space etc.). We compare house characteristics in our sample to the full BHPS sample. On average we do not find large differences in the number of rooms, type of central heating, council tax, although 94 % of houses in the BHPS have a garden against 91 % in our sample.

In Panel (c) of Table 1 we report selected descriptive statistics of the potential mechanisms. The proportion of people paying for supplementary private insurance is very low. On average, respondents go to the doctor about three times a year. Approximately 43 % are smokers and the smokers smoke an average of 16 cigarettes per day. About 43 % of our estimation sample are employed, work about 34 h a week and spend about 20 min per day travelling to work. Compared to the full BHPS sample, the sample of initial public renters were less likely to have a garden, more likely to live in polluted areas and in areas with vandalism problems. In Appendix C (in Supplementary material) we examine the quality of our house price predictions by considering several assumptions and distributional properties.

### 6.2 Macro-level results

Higher Right to Buy discounts are associated with higher levels of home ownership (column (1); Table 2). The first-stage F-statistic is 17.02 implying that at a LAD-level, the Right to Buy discount is a strong predictor of ownership rates and hence our instrument is relevant (Stock and Yogo, 2005; Stock et al., 2002).

**Table 2:** Local Authority District level fixed-effects instrumental variables analysis of home ownership on health.

	(1)	(2)	(3)
	(First stage)	(Second stage)	(Second stage)
	LAD ownership rate	Rate of people with LHC	Average no. of health probs.
LAD ownership rates		−0.198***	−0.493***
(*instrumented*)		(0.053)	(0.0151)

Max. RtB discount (£'000 s)	0.0024***		
(*instrument*)	(0.000)		

% of population aged 65+	−0.0124***	0.00931***	0.0181***
	(0.002)	(0.002)	(0.006)

Median weekly pay (deflated)	0.00000146	−0.000025	−0.000014
	(0.000)	(0.000)	(0.000)

Median hours worked per week	0.00308	−0.00310*	−0.00650
	(0.002)	(0.002)	(0.005)

% of population economically active	0.00164***	−0.00185***	−0.00620***
	(0.000)	(0.000)	(0.001)

Year dummies	Yes	Yes	Yes

First-stage F-statistic	17.02		
Observations (N*T)	2161	2161	2161

Sample includes initial public renters only, 2003 – 2010. We have information on N = 311 LADs. Standard errors in parentheses. * p < 0.10, ** p < 0.05, *** p < 0.01. LHC = longstanding health condition (lasting at least 12 months).

LADs with higher home ownership (instrumented by Right to Buy discounts) have lower rates of longstanding health conditions; a 10-percentage point increase in the home ownership rate reduces the rate of people with longstanding health conditions by about 2 percentage points. Similarly, higher home ownership is associated with lower numbers of health problems; a 10-percentage point increase in the home ownership rate reduces the average number of health problems by around 0.5.

### 6.3 Individual-level results

#### 6.3.1 First stage results: how Right to Buy discounts affect the probability of home ownership

The Right to Buy discount is a statistically significant predictor of home ownership uptake; a £10,000 increase in the Right to Buy discount increases the probability of ownership by 2 (= 0.002*100*10) percentage points (Table A3 in Appendix A.3 (in Supplementary material)). The first-stage Likelihood-Ratio (LR)-statistic is 1036.67, meaning that the Right to Buy discount is a very strong predictor of ownership and hence our instrument is relevant (Stock and Yogo, 2005; Stock et al., 2002).

The longer an individual has lived in their publicly rented property, the less likely they are to buy it; every additional year in the property reduces the probability of ownership by 0.1 percentage points. Also, people who live in areas with expensive average house prices, ceteris paribus, are less likely to buy; a 10 % increase in average local property prices reduced the probability that an individual becomes an owner by 0.8 percentage points.

#### 6.3.2 Second Stage Results: how home ownership affects health and well-being

We report the second stage results along with one stage model estimates in Table 3. Homeownership is associated with higher self-assessed health in both the one and two stage models. The significance of the first stage residual indicates it is necessary to account for endogeneity. Being an owner increases self-assessed health by 0.19 points on a five-point scale.

**Table 3:** Single-stage and second-stage models of physical and psychological health outcomes.

	(1)	(2)	(3)	(4)	(5)	(6)
Outcome:	SAH		GHQ		#Probs	
Model:	OLS	2SRI OLS	OLS	2SRI OLS	Nbreg	2SRI Nbreg
Being a home owner	0.248***	0.185*	0.455	1.458**	−0.441***	−0.651***
	(0.048)	(0.104)	(0.287)	(0.587)	(0.088)	(0.149)

First Stage Residual		0.023***		−0.365**		0.074**
		(0.003)		(0.151)		(0.035)

Socioeconomic Characteristics^a^	Yes	Yes	Yes	Yes	Yes	Yes

Housing Characteristics^b^	Yes	Yes	Yes	Yes	Yes	Yes

Year x Region^c^ Interactions	Yes	Yes	Yes	Yes	Yes	Yes

Observations (N*T)	6430	6430	6430	6430	6430	6430

Sample includes initial social renters only, 2000 – 2008. Coefficients displayed Bootstrapped standard errors, based on 2000 replications, in parentheses. * p < 0.10, ** p < 0.05, *** p < 0.01.

The estimates presented in columns (1) - (4) are coefficients. The estimates presented in columns (5) and (6) are marginal effects, calculated at the means of independent variables.

SAH = self-assessed health; GHQ = General Health Questionnaire; #Probs.=Number of self-reported health conditions. OLS = ordinary least squares; 2SRI = two-stage residual inclusion; Nbreg = negative binomial regression.

a Additional controls include: sex, age, age squared, log of monthly household income, marital status, and educational attainment.

b Housing characteristics included are: heating type, problems with vandalism or crime, problems with pollution, whether there is a garden.

c England is broken down into 16 regions. We include regions as opposed to lad dummies as using lower levels of geography encountered problems with collinearity.

When we consider GHQ as an outcome, we can see there is no effect of homeownership in a one-stage OLS model (column (3)). However, when we consider a two-stage model (column (4)), we see that the effect of predicted homeownership on GHQ is large in magnitude (β=1.4) and statistically significant. As the endogeneity test (first stage residual) rejects the null hypothesis that home ownership is exogenous, we prefer the two-stage model results.

Home ownership is associated with a reduction in the number of chronic conditions in both the one-stage and two-stage models. The reduction is larger in the two-stage model (0.65 compared to 0.44), and our preferred model is the two-stage model, due to the significance of the first stage residual.

In separate models, which we do not present here, we change the set of control variables in the models reported in Table 3. Separately, we additionally include more household characteristics that may change as a result of ownership (i.e. the number of rooms) and the number of children present in the household. The main results are qualitatively very similar and are available on request. We do not present them here as it could be argued that having children, for example, would be endogenous to either home ownership decisions or health, or even both.

### 6.4 Robustness checks

The relationships between ownership and health and well-being outcomes for private renters are not statistically significant. The coefficients maintain the expected direction but are much smaller than for public renters.

The results are qualitatively and quantitatively very similar when we remove the outlier values of Manchester, Birmingham, West Devon and South Buckinghamshire from the macro-level analysis (results available on request).

**Fig. 2:**
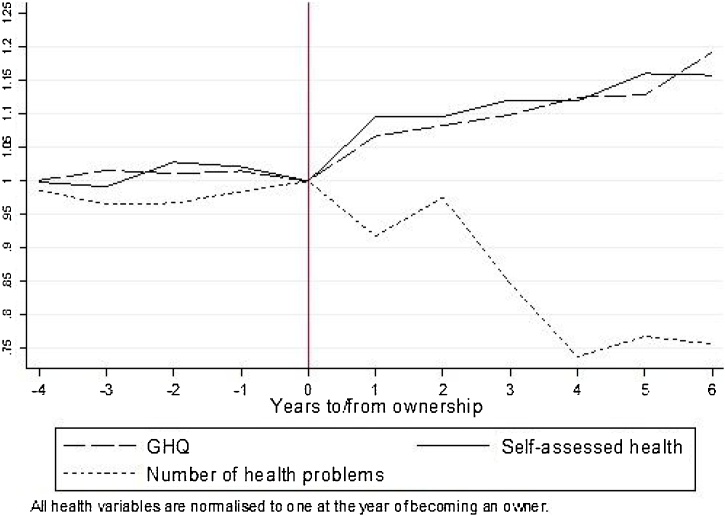
Average health for those who become an owner by years to/from ownership.

Source: Authors' representation from BHPS data 1999–2008.

To descriptively examine the potential for lead and lag effects at the individual level, we plot the average health status of those who do go on to become a home owner against years to and from becoming an owner in Fig. 2. For graphical clarity, we normalise all health measures to one at the year ownership occurs. We focus on four years prior to and six years post ownership, to ensure all cells contain at least 35 observations.

There are no anticipation effects. In the four years leading up to ownership all three health measures appear constant. However, after becoming an owner there are marked improvements in all three health measures. GHQ and SAH increase and there is a reduction in the number of health problems reported. This suggests that there are improvements in the health of owners rather than deteriorations in health of those who continue to rent.

The results from the remaining robustness checks are available in Appendix B (in Supplementary material). They show that the findings are robust.

### 6.5 Selection into home ownership

If healthier initial renters are more or less likely to go onto purchase the homes they live in, we could have issues with identification brought about through a ‘selection effect’. In order to investigate if there is a selection effect into home ownership, we regress a binary indicator of whether an individual will become a home owner against baseline health (reported the first time an individual is observed in the data) as well as other possible confounding variables (as reported in notes a and c of Table 3). We observe that initial health has no statistically significant effect on predicting future home ownership (Table B4 (in Supplementary material)). We would like to acknowledge an anonymous referee for this suggestion.

## 7 Mechanisms

Investigating the mechanisms through which home ownership affects health is important for the design of policies that can influence these pathways. In the BHPS there is limited availability of specific inputs of the health production function. For instance, we have no information on food expenditure, or the time spent on leisure activities. Nevertheless, using the limited data available we explore some potential mechanisms (Table 4).

**Table 4:** Second-stage models of physical and psychological health outcomes for first robustness check (placebo policy of private renters).

	(1)	(2)	(3)
Outcome:	SAH	GHQ	#Probs
Model:	2SRI OLS	2SRI OLS	2SRI Nbreg
Being a home owner	0.099	0.458	−0.277
	(0.125)	(0.458)	(0.173)

First stage residual	−0.003	−0.079	−0.033
	(0.044)	(0.263)	(0.059)

Sample includes initial private renters only, 2000−2008. Initial private renters are matched to similar initial public renters using propensity score matching. We have information on N = 545 individuals. Bootstrapped standard errors, based on 2000 replications in parentheses. * p < 0.10, ** p < 0.05, *** p < 0.01. Marginal effects, from second stage models, calculated at the means of independent variables. The models contain all additional variables as reported in Table 3.

First, we follow Apouey and Clark (2015) and examine the effect of home ownership on different components of health. The purpose of this exercise is twofold. Firstly, the temporal dimension of health and its measurement (Mullahy, 2016) has implications as to whether housing policies could have long-lasting or shortly-lived health effects. In the Grossman (1972) human capital model there is a distinction between health capital (i.e. a stock measure of health) and health status (i.e. “healthy time” or flow measure of health). For instance, in the BHPS the self-assessed health variable is anchored to a time dimension asking respondents to rate their health in the past 12 months. As such, it can be considered a flow measure. Instead, specific categories of health are not attached to any time dimensions and are therefore measures of the stock of health^2^ . Secondly, the type of condition might inform us of the potential pathways between home ownership and health. For instance, if diabetes^3^ is affected by home ownership, we might expect potential pathways to be through lifestyle behaviours as being overweight, unhealthy diet and physical inactivity are three of the major risk factors for type 2 diabetes (World Health Organization, 2016). Therefore, we modify Eq. (4) where *H* indicates each of the six dummies of health conditions. We find that home ownership is associated with a 14-percentage point lower probability of reporting cardiovascular conditions (Table 5).

**Table 5:** Second-stage models of mechanisms.

Panel (a): Specific health conditions						
	(1a)	(2a)	(3a)	(4a)	(5a)	(6a)
	Musculoskeletal	CVD	Skin problems	Respiratory problems	Depression	Other chronic
Being a home owner	0.0311	−0.1365***	−0.1485***	−0.1289***	−0.0166	−0.0357
	(0.0526)	(0.0393)	(0.0391)	(0.0362)	(0.0327)	(0.0335)
First stage residual	−0.0421**	0.0072	0.0315***	0.0187*	−0.0017	0.006
	(0.0175)	(0.0111)	(0.0615)	(0.0109)	(0.0103)	(0.0101)
Observations (N*T)	6430	6430	6430	6430	6430	6430

Panel (b): Health seeking behaviours						
	(1b)	(2b)	(3b)	(4b)	(5b)	

	Private health insurance	No. visits to the doctor	No. cigarettes	Smoker	Active	

Being a home owner	0.0414**	−1.6989***	−0.4391	−0.1075**	0.0622	
	(0.0194)	(0.2872)	(1.2602)	(0.0466)	(0.0625)	
First stage residual	−0.0011	0.2078	0.2377	0.0105	−0.0109	
	(0.0067)	(0.0922)	(0.4235)	(0.0155)	(0.0188)	
Observations (N*T)	3903	6419	2751	6430	2954	

Panel (c): Labour market and economic resources						
	(1c)	(2c)	(3c)	(4c)	(5c)	

	Employed	Working time	Commuting time	Expenditure on leisure	Housing costs	
Being a home owner	0.1114**	−2.5493	−5.4086*	6.5273**	200.846***	
	(0.0535)	(1.5348)	(2.6990)	(2.7736)	(12.7978)	
First stage residual	0.0484***	1.5743***	1.7619*	1.2641	−24.4117**	
	(0.0162)	(0.5582)	(1.7619)	(0.8905)	(4.1067)	
Observations (N*T)	6414	2707	2511	6401	6404	

Panel (d): Housing quality						
	(1d)	(2d)	(3d)	(4d)		

	Gas/electricity	Garden	Pollution	Vandalism		
Being a home owner	−0.0254	0.0632**	−0.086	0.0249		
	(0.0254)	(0.0335)	(0.0327)	(0.0397)		
First stage residual	−0.0004	0.0034	0.0024	−0.0196		
	(0.0079)	(0.0112)	(0.0114)	(0.0132)		
Observations (N*T)	6424	5834	5879	6424		

Panel (e): Social capital						
	(1e)	(2e)	(3e)			

	Vote	Talk to neighbours	Satisfaction with			
			home			

Being a home owner	−0.0024	−0.7482***	0.3808**			
	(0.0382)	(0.2461)	(0.1311)			
First stage residual	−0.0012	0.1157	0.0009			
	(0.0123)	(0.0861)	(0.0428)			
Observations (N*T)	6426	6399	5564			

Each panel contains a separate second stage model. Sample includes initial public renters only, 2000 – 2008. Bootstrapped standard errors, based on 2000 replications, in parentheses. * p < 0.10, ** p < 0.05, *** p < 0.01. Marginal effects, from second stage models, calculated at the means of independent variables. The models contain all additional variables as reported in Table 3.

Second, we directly observe some of the inputs of the health production function such as risky health behaviours, number of visits to the doctor and the purchase of private medical insurance. We expect home ownership to have an ambiguous effect on these. On the one hand, there is a wealth effect, meaning those who become home owners might be able to extract equity from their house and spend it on goods such as alcohol or cigarettes. If these are normal goods, home ownership might have detrimental effects on health. However, the wealth effect might allow individuals to purchase private medical care and have quicker access to treatment thereby improving health. On the other hand, there might be a time effect. Individuals might reduce their working hours by substituting wages for the equity extracted from the house. This extra time might be spent on preventive activities or on leisure activities (healthy or unhealthy). The extent to which individuals invest in healthier lifestyle behaviours might depend on their time preferences. Home ownership might change the intertemporal trade-off between current and future outcomes shifting individual preferences towards the future when the house can be fully owned or more equity can be extracted. In this case, individuals have more incentive to invest in their health. Or else more forward-looking individuals become home owners and invest more in their health. We explore these factors by modifying Eq. (4) and estimating a series of models of private health insurance, the probability to become a smoker and being active, and linear models for the number of visits to the doctor and number of cigarettes smoked by smokers. We find that those who become home owners are 11 percentage points less likely to smoke (column 4b). This result might also explain the changes in lifestyle-related conditions such as CVD. With regards to health-seeking behaviours (panel (b) of Table 5), those who become home owners are four percentage points more likely to buy private health insurance and go to the doctor about two times fewer per year than renters.

A third channel, Disney and Luo (2017) suggest that although the Right to Buy increased home ownership, this came at the expense of housing quality. The supply of accommodations eligible for the Right to Buy, although cheaper, tended to be of poor quality. There is evidence of a detrimental effect of poor house quality on health (Shaw, 2004; Marmot et al., 2008). We do not have detailed information on the quality of the house in the BHPS. However, we have some information on the characteristics of the house that might directly impact on health. We modify Eq. (4) to separately estimate four models where the dependent variable is equal to one if the house where individual i lives has central heating, if it has garden, if there are issues with pollution or if there are issues with crime/vandalism. Note that because an individual who becomes an owner as a result of the Right to Buy scheme cannot move, we infer that they have redeveloped some land to create a garden (changing a yard to a garden, say) and their perceptions of their local area have changed, rather than actual observable changes to their local area. One individual becoming a home owner is unlikely to reduce area level pollution, say, but could impact on that individual's perceptions. We find that those who become home owners are 13 percentage points less likely to report respiratory problems and seven percentage points more likely to have a garden. As noted above, as household location is fixed for people who bought under the policy, this suggests that people redeveloped existing land into a usable garden space. We explore the possibility of excluding potential movers in Appendix B (in Supplementary material) to further strengthen this claim. We do not find evidence of changes in pollution or vandalism as a result of an increase in home ownership.

A fourth potential channel is via labour market activities. We investigate whether home ownership is positively associated with the likelihood of becoming employed and whether employed people change their working hours in response to the policy. We do so, by using a two-part model for Eq. (4). First, we use a logit model where the dependent variable indicates whether individual i is employed. Then we use a linear model where the dependent variable is working time measured with hours per week (if individual i is employed). These labour market consequences of the policy might have ambiguous effects on health depending on the relative size of the substitution and income effects. We find that those who become home owners are 11 percentage points more likely to be employed.

A fifth channel is via non-market time activities and economic resources. If home ownership and commuting times are negatively related, then individuals might spend this extra time in the production of health. However, it is worth noting here that individuals who exploit the Right to Buy policy by definition cannot move their home location. Therefore, any change to commuting distance must be brought about by changes in workplace location, travel mode, or transport infrastructure (Munford et al., 2015). We estimate Eq. (4) with a linear model of commuting time (for those in employment). There is also a wealth effect as home owners can extract equity from their house or have more resources if their mortgage is lower than their rent, or if they were able to buy outright and are now rent-free. These extra resources could be used on leisure activities which we capture by estimating Eq. (4) with a linear model on expenditure on leisure activities measured in pound sterling per month. The BHPS records information on housing costs either in the form of monthly rental or mortgage payments. We modify Eq. (4) to be a linear model of housing costs. Although we find no evidence of a reduction in working time (Fichera and Gathergood, 2016), we find they spend about 5 min less travelling to work than renters. Our results suggest that they spend the extra resources (from working or saving on rent) and, to some extent, the extra time available to spend about six extra pounds on leisure activities. When we consider total monthly household costs, we find that those who own their home spend approximately £200 more a month on their home that those who rent.

Finally, the social capital channel may operate via increased political participation, the building of social ties with neighbours and increased satisfaction with the home which home owners can improve (Fischer, 1982; Glaeser and Sacerdote, 2000). Therefore, we estimate Eq. (4) with linear models of voting behaviour, of a variable indicating whether the BHPS respondent talks to their neighbours, and their satisfaction with their home. Our results suggest that owners are less likely to talk to their neighbours and are much more satisfied with their home than renters. This might be related to the time effects we find, where there is no change in working hours but an increase in expenditure on leisure activities which may impact on the time spent talking to neighbours. There is no difference in voting behaviour between renters and owners.

## 8 Discussion

We find that the Right to Buy scheme led to increased levels of home ownership, and that this home ownership is associated with better levels of health, both at the macro (area) and micro (individual) level. The results are consistent across both subjective and more objective measures of health and robust to several additional checks including falsification tests and exclusion of potential outliers. The Right to Buy policy has previously been shown to be a success in that it encourages individuals to buy their home. To our knowledge, this is the first study that quantifies the effect it had on health and well-being.

The magnitude of the estimated effects is reasonable as previous studies have found that unemployment reduces self-assessed health by 0.23 points (Böckerman and Ilmakunnas, 2009) and reduces GHQ by between 0.83 and 2.2 units, depending on the GHQ scale (Clark, 2003; Wildman and Jones, 2002; Flint et al., 2013). This reduction is comparable to the results we have presented here; becoming unemployed is at least as ‘bad’ for health as becoming a home owner is ‘good’.

When considering the mechanisms behind our results, our models suggest that these operate via the labour markets with new job opportunities (conditional on a fixed household location), extra time saved travelling and resources available for (healthy) leisure activities. We also find evidence to suggest that those who go onto become owners are less likely to have unhealthy behaviours (such as smoking) and less likely to suffer from cardiovascular and respiratory conditions. Those who become owners are also more likely to buy health insurance and make fewer visits to their GP.

There are several limitations to our analysis. First, we have only looked at the benefits to the people who were eligible and our findings do not constitute a full population evaluation. Second, we do not know whether those individuals who were eligible for the Right to Buy scheme and then went onto become an owner took advantage of the scheme. Third, we have considered home ownership as the main effect of the Right to Buy policy. This can be thought of as the ‘extensive’ margin. It may be interesting to consider the ‘intensive’ margin and look at the wealth (and/or income) effects alongside home ownership but we cannot do so with the data available.

Our finding that home ownership has a positive impact on health, might support initiatives such as the Affordable Homes Programme being implemented in the UK. Our results support Buck et al. (2016) suggesting that population health cannot be improved by the National Health Service (NHS) alone and that appropriate housing policies, such as affordable housing, can support health policies (NHS England, 2014). Also, because we find that some mechanisms operate via reduced travelling time and extra time spent on healthy leisure activities, improvements in the infrastructure and transport system that reduce travelling time might also be beneficial to health. More widely, our findings support the idea that, as health is determined by wider socio-economic factors, non-health policies can impact on health.

## CRediT authorship contribution statement

**Luke A. Munford:** Conceptualization, Methodology, Formal analysis, Data curation, Writing - original draft, Writing - review & editing, Funding acquisition. **Eleonora Fichera:** Conceptualization, Methodology, Writing - original draft, Writing - review & editing, Supervision. **Matt Sutton:** Conceptualization, Writing - review & editing, Supervision.

## Declaration of Competing Interest

None.

## Footnotes

1 Council tax, introduced in 1993, is a tax on domestic properties in England. Every property is placed into one of eight bands, depending on the assumed capital value of the property as of 1st April, 1991. Properties constructed after 1991 are assigned a nominal assumed capital value, based on 1991 prices.

2 Although we can expect chronic conditions to be less transitory than conditions such as back problems, we have no information on the time-span they occur in.

3 However, we note that we do not have information on the type of reported diabetes.
