# Supplementary material for: Is owning your home good for your health? Evidence from exogenous variations in subsidies in England
Source: Econ Hum Biol. 2020 Dec;39:100903. doi: 10.1016/j.ehb.2020.100903 (PMC7725589; doi:10.1016/j.ehb.2020.100903)
Supplement: Supplementary file 2 [file mmc2.docx]

**Appendix A: Further details on data**

**A.1 Macro-level data**

Table A1 presents information on the data, along with its source, used in the macro (LAD)-level analysis.

**Table A1:** Description of data used in the macro-level analysis

| **Variable** | **Definition** | **Source** |
| --- | --- | --- |
| Home ownership rate | The proportion of people in a LAD who report that they own (outright or through a mortgage) their home | Authors derivations from LFS data. The variable we use is ‘TEN1'. ^a^ |
| RtB Sales | The number of houses sold through the RtB scheme in each LAD | Department for Communities and Local Government (DCLG). Table 685, available online. ^b^ |
| RtB maximum discount | The maximum available RtB discount in each LAD | Email communication from DCLG. |
| % of population who report having a longstanding health condition | The average number of people who report having a longstanding health condition (LHC) in a LAD. A LHC is defined as lasting more than 12 months. | Authors derivations from LFS data. The variable we use is ‘LNGLIM’. ^c^ |
| Average number of (self-reported) health conditions | The average number of self-reported health conditions individuals in a LAD have. | Authors derivations from LFS data. Individuals are shown a list of conditions and indicate all that they have. |
| % who are economically active | The percentage of the LAD population who report they are economically active (including the employed and the unemployed who are actively seeking work). | NOMIS. ^d^ |
| Median hours worked per week | The median number of hours worked by an individual per week in a LAD. | NOMIS. ^d^ |
| Median weekly pay | The median weekly salary received by an individual in a LAD (deflated using the RPI with 2008 as the base year). | NOMIS. ^d^ |
| Population size | The total size of the LAD; all ages. | NOMIS. ^d^ |
| % of population who are aged 65 years and older | The proportion of individuals in an LAD who are aged 65 years and above. | NOMIS. ^d^ |
|  |  |  |
| Notes:  a: we define owners as responses (1) owned outright and (2) being bought with a mortgage or loan.  b: <https://www.gov.uk/government/statistical-data-sets/live-tables-on-social-housing-sales>  c: we are aware that this variable changed in Spring 2000 (in that it was asked to more individuals; previous to this it was limited to individuals of working age only, whereas after spring 2000 it was asked to all respondents of working age or those aged 75 and under and first contact or those aged 75 and over and are not too ill/distressed to continue) and again in Spring 2013 (being replaced with ‘LNGLST'). However, these changes do not affect our time frame of consideration (2003 – 2010).  d: NOMIS is the Office for national Statistics (ONS) official labour market statistics portal – see <https://www.nomisweb.co.uk/> | | |

Table A2 presents regression output from models where we examine for associations between the maximum value of the Right to Buy discount cap and local levels of health.

| **Table A2**: Local Authority District level models of the relationship between the maximum Right to Buy discount cap and other factors | | | |
| --- | --- | --- | --- |
|  | (1) | (2) | (3) |
|  | Pooled | Fixed-effects | Pooled with one period lag |
| Rate of people with LHC | 5.528 | 7.150 | 3.135 |
|  | (6.447) | (4.953) | (7.692) |
|  |  |  |  |
| Average no. of health probs. | 0.845 | 1.418 | 2.207 |
|  | (2.146) | (1.599) | (2.652) |
|  |  |  |  |
| % of population aged 65+ | 0.792*** | 1.165* | 0.969*** |
|  | (0.146) | (0.486) | (0.175) |
|  |  |  |  |
| Median weekly pay (deflated) | 0.008 | -0.002 | 0.010 |
|  | (0.005) | (0.006) | (0.006) |
|  |  |  |  |
| Median hours worked per week | -0.397 | 0.011 | -0.405 |
|  | (0.229) | (0.201) | (0.268) |
|  |  |  |  |
| % of population economically active | 0.069 | -0.033 | 0.096 |
|  | (0.052) | (0.038) | (0.068) |
|  |  |  |  |
| % of population with no qualifications | 0.063 | 0.044 | 0.089 |
|  | (0.049) | (0.044) | (0.058) |
|  |  |  |  |
| Crime rate per 1,000 population | -0.086*** | 0.122*** | -0.102*** |
|  | (0.015) | (0.023) | (0.017) |
|  |  |  |  |
| 2001 Census information | Yes | No | Yes |
|  |  |  |  |
|  |  |  |  |
| Year dummies | Yes | Yes | Yes |
|  |  |  |  |
| Observations (N*T) | 2161 | 2161 | 1866 |
| Sample includes initial public renters only, 2003 – 2010. We have information on N=311 LADs. Standard errors in parentheses. * p<0.10, ** p<0.05, *** p<0.01. information from the 2001 Census includes: % of properties owned outright, % of properties owned with a mortgage, % of property socially rented, % of properties privately rented, and IMD rank of median LSOA in LAD. LHC=longstanding health condition (lasting at least 12 months); IMD=index of multiple deprivation, LAD=local authority district; LSOA=lower super output area. | | | |

**A.2 Individual level data in the BHPS**

**A2.1 Socioeconomic and demographic variables at the individual level**

As well as detailed housing information, the BHPS contains a wealth of information about the demographic characteristics and socioeconomic position of each respondent, including gender, age, marital status, highest educational qualification attained, number of people who live in the household, and equivalised monthly household income. We use age in years (and its squared value). Gender is self-reported, and we include a dummy variable equal to one if the respondent replies they are male, zero otherwise. We use information on present legal marital status (“*What is your current legal marital status, are you…*” and a list of nine options is given), creating a dummy variable equal to one if the response is either married (including cohabiting) or in a civil partnership, zero otherwise. For education, we use a question which asks about highest academic qualification, and we create three dummy variables: one for university level education (including undergraduate and postgraduate), one for college level qualifications (including A-levels), and one for school level qualifications. The omitted category is ‘no qualifications’.

There is consistent international evidence that amongst the dimensions of socioeconomic status education is the key determinant of health (Cutler et al., 2008; Cutler and Lleras-Muney, 2010). We consider the highest educational attainment because it appears to be the strongest predictor of mortality rather than years of schooling which might capture individuals repeating school (Clark and Royer, 2013).

For income, we use a measure of total household weekly net income, which has been equivalised (using the OECD equivalence scale) and deflated for inflation. This is a derived variable (*hhnetde2*) available in the BHPS Derived Current and Net Household Income Variables dataset. To generate monthly income data, we multiply the value by 52 and then divide by 12. We additionally use information on the number of people who live in the household, which we include as a continuous variable.

The relationship between family size and health is ambiguous. On the one hand, according to the quantity-quality model an increase in family size is associated with unhealthier children because family size is an input in the health production function and the cost of investing and increasing the health of children increases with the size of the family (Becker and Lewis, 1973). On the other hand, empirical evidence finds the opposite, suggesting that in smaller families children are less exposed to diseases and this in turn weakens the development of their immune system (Karmaus and Botezan, 2002; Bevier et al., 2011). The spousal health literature suggests an ambiguous relation between individual health and that of her partner. On the one hand, “social contagion’’ implies that individual outcomes change the marginal utility of the partner’s actions. Social comparisons between spouses, for example, attenuate the negative impact of unhealthy outcomes (i.e. weight). However, the healthcare needs that these outcomes require might incentivise the partner to improve her health. Using German panel data Clark and Etilé (2011) find evidence supporting the social contagion hypothesis. However, this is not to be interpreted as causal evidence because of the lack of exogenous variation in spousal health. We consider the total number of people in the household comprising both adults and children.

**A2.2 Labour, leisure and housing costs**

In the mechanisms analyses, we consider several labour market and leisure outcomes. We define “employed” as a dummy variable equal to one if the respondent is employed or self-employed, zero otherwise, including unemployed and retired. For those respondents who are in work, we consider working time which indicates the number of hours worked per week and commuting time which is the number of minutes spent travelling to work, one-way per day. BHPS respondents are asked the amount (in pound sterling) of expenditure on leisure activities per month from an eleven point-interval list of values ranging from under £10 to £160 or over. We take the midpoint value of each interval and treat this variable as continuous. We also consider the housing costs that a BHPS respondent incurs for either mortgage or renting. We make use of the BHPS derived variable “*xphsn*” which is defined as “*net monthly mortgage or rent costs. For renters who receive housing benefit, either partial or complete, includes the rent after the rebate. Variable is zero for houses rent free or owned outright*”. As a robustness check, we compute similar variables ourselves, based on reported rent and mortgage payments, and the results were very similar.

**A2.3 Social capital**

For the analysis of social mechanisms, we take a broad definition of social capital and consider measures of political and social participation, and of satisfaction. Although the BHPS is rich in measures of social activity and political participation, our choice of variables is restricted to those that were available across many years and that displayed sufficient variation in our sample of interest. We define “*vote*” as a dummy variable equal to one if the respondent supports a particular party. The variable “*Talk to neighbours*” is a dummy that equals one if the respondent talks to neighbours at least once or twice a week. “*Satisfaction with home*” is a score that indicates how satisfied the respondent is with her home and it goes from one (not satisfied at all) to seven (completely satisfied).

**A.3 First stage output**

| **Table A3**: First Stage model of hypothetical Right to Buy discount on ownership | | |
| --- | --- | --- |
|  | (1) | (2) |
|  | Coefficient | Marginal Effect# |
|  |  |  |
|  |  |  |
| Calculated RtB disc./1000 | 0.0266*** | 0.002*** |
|  | (0.006) | (0.0003) |
|  |  |  |
| Time lived at property (years) | -0.0184*** | -0.001*** |
|  | (0.008) | (0.0004) |
|  |  |  |
| Log(average LAD house price) | -1.139*** | -0.081*** |
|  | (0.164) | (0.0088) |
|  |  |  |
| Socioeconomic Characteristics a | Yes | Yes |
|  |  |  |
| Year dummies | Yes | Yes |
|  |  |  |
| Observations | 6430 | 6430 |
| Sample includes initial social renters only, 2000 – 2008. Coefficients displayed. Bootstrapped standard errors, based on 2,000 replications, in parentheses * p<0.10, ** p<0.05, *** p<0.01 | | |
| a: additional controls include: sex, age, age squared, log of monthly household income, marital status, and educational attainment.  #: Marginal effects calculated at the means of independent variables. | | |
|  | | |

**References for Appendix A**

Becker, G. S. and H. G. Lewis (1973). On the interaction between the quantity and quality of

children. Journal of Political Economy 81(2, Part 2), S279-S288.

Bevier, M., M. Weires, H. Thomsen, J. Sundquist, and K. Hemminki (2011). Influence of family size and birth order on risk of cancer: a population-based study. BMC Cancer 11(1), 163.

Clark, A. E. and F. Etilé (2011). Happy house: Spousal weight and individual well-being. Journal of Health Economics 30(5), 1124-1136.

Clark, D. and H. Royer (2013). The effect of education on adult mortality and health: Evidence from Britain. The American Economic Review 103(6), 2087-2120.

Cutler, D. M. and A. Lleras-Muney (2010). Understanding differences in health behaviours by education. Journal of Health Economics 29(1), 1-28.

Cutler, D. M., A. Lleras-Muney, and T. Vogl (2008). Socioeconomic status and health: dimensions and mechanisms. Technical report, National Bureau of Economic Research.

Karmaus, W. and C. Botezan (2002). Does a higher number of siblings protect against the development of allergy and asthma? a review. Journal of Epidemiology & Community Health 56(3), 209-217.

**Appendix B: Remaining robustness checks**

**B.1 Robustness checks for main analysis**

**B.1.1 How to calculate the estimated value of a rented property**

In the reduced specification of the house price equation, the coefficient on local average house prices is 1.25 (standard error = 0.011; t-statistic = 115.66). This is larger than the corresponding coefficient in the full model (0.94; first row of Table C.1 in Appendix C). The adjusted R-squared of the model with only local average house prices is 24%, compared to the adjusted R-squared in the full model of 65%.

In Figure B1 we show the relationship between the two predictions, and we see this is upward sloping and close to the 45-degree line. However, there is some variability, and in Figure B2 we plot the distribution of the predicted values from the full model (grey bars with no lines) and the reduced model (clear bars with black lines). For graphical quality, we have censored the upper tail at £1,000,000. This figure shows that the two distributions are quite similar, with the reduced model’s predictions being slightly to the right of the full model’s, on average. This indicates, as in Benítez-Silva et al. (2015), that the value of cheaper properties is over-estimated compared to the average price in the same LAD.

When turning to the results of the second stage equation, in panel (a) of Table B1 we observe that how we predict house prices for public renters makes very little qualitative difference in that the sign and significance is the same. The magnitude of the coefficients is also quite similar.

**B.1.2 Exclude income**

When we remove income from the set of control variables (panel (b), Table B1) we observe that the main result holds true; home ownership is good for health. However, the magnitude of the relationship is slightly smaller for all three health measures when we exclude income.

**B.1.3 Consider the head of household and non-head of household separately**

In our data, we have 4,185 observations on 858 individuals who are classified as being the head of household (as defined in the BHPS. The BHPS definition of the head of household is defined as the principal owner or renter of the property, and (where there is more than one), the eldest taking precedence.). The results for GHQ and the number of conditions are qualitatively the same as the main results (columns (2) and (3), panel (c) of Table B1). However, when we consider self-assessed health as the outcome, we observe no statistically significant effect.

We additionally have 2,245 observations on 598 individuals who are classified as not being the head of household. (Note that the number of observations between the two subsample (4185+2245) sums to the full sample (6430). However, the number of individuals in the two subsamples (858+598=1456) is larger than the overall number of observations (1204). This is due to the fact that head of household is not stable over time; for example, an individual maybe the head of household in one wave, but not the next.) For the non-heads (panel (d) of Table B1) we observe consistently larger health benefits than reported in the main results (Table 3) and for the subsample of heads of household. We attribute this to the fact that the decision to become a home owner may have been exogenously imposed upon the non-heads by the head. It is also possible to imagine that non-heads face less financial pressure, and hence enjoy living in an owned home more.

**B.1.4 Consider only people who are employed when first observed**

We have information on 527 individuals (2,790 observations) who meet our definition of being initially employed. The results based on this sample are qualitatively similar to the main results (panel (e) of Table B1); we observe the same sign and statistical significance. The effects on self-assessed health and the number of conditions are slightly smaller, but larger for GHQ in this sub-sample. We therefore conclude that our main results are not being driven by the policy being taken up more by individuals in employment compared to individuals who are unemployed or retired.

**B1.5 Function form of second stage**

Given that self-assessed health is technically an ordinal variable, we estimate marginal effects following an ordered logit regression (Table B2), where we observe for both the one- and two-stage models, that ownership increases the probability of being in the top two responses (excellent and very good) and reduces the probability of being in the bottom three classes (good, fair, and poor). We prefer the two-stage results, due to the significance of the first-stage residual in the second stage outcome model.

**B.2 Robustness checks for mechanisms**

Some of the potential mechanisms we explore above could be interpreted as an individual owning a different house to the one they previously rented (e.g. the garden mechanism and changes in commuting time). To attempt to establish if the individual bought the house they were renting (and hence more likely to exploit the Right to Buy scheme), we perform a final robustness check. Through a special license version of the BHPS, we are able to obtain the Lower Super Output Area (LSOA) of each respondent. LSOAs are small levels of administrative geography based on the 2001 Census. Each LSOA contains approximately 1,500 individuals. For the purpose of this robustness check, we assume that if an individual does not change their LSOA throughout their time in the sample, then they have not moved house. Moves within an LSOA are uncommon due to their small size. Based on this constant LSOA assumption, our sample reduces from N= 1204 [NT=6430] to N=983 (82%) [NT=3908; 61\%]. We then repeat the analysis on this subsample, and the results are reported in Table B3. We observe that the results are essentially the same in terms of direction and statistical significance. We have additionally performed robustness checks on variables we infer could also be proxies for constant household location and the results remain robust (available from the authors on request). We therefore conclude that our results are not impacted upon by people moving to (and buying) houses other than the ones they were previously renting; that is, we can make stronger clams regarding the likely impact of the Right to Buy scheme.

**Figures and Tables for Appendix B**

**Figure B1:** The relationship between the two predicted values: full model and reduced model


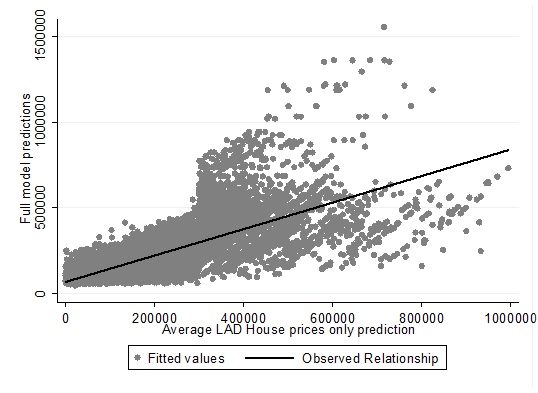


*Source: authors’ representation from BHPS data 1999-2008.*

**Figure B2:** Comparing the distribution of predicted house values


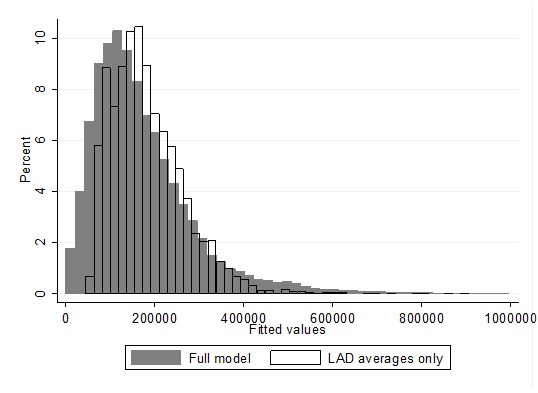


*Source: authors’ representation from BHPS data 1999-2008. For graphical clarity, we censor the observations at £1,000,000.*

| **Table B1:** Second-stage models of physical and psychological health outcomes for remaining robustness checks | | | | |
| --- | --- | --- | --- | --- |
|  |  | (1) | (2) | (3) |
|  | Outcome: | SAH | GHQ | #Probs |
|  | Model: | 2SRI OLS | 2SRI OLS | 2SRI Nbreg |
|  |  |  |  |  |
| Panel (a) | Being a home owner | 0.194** | 1.429*** | -0.658*** |
| Only LAD averages |  | (0.082) | (0.489) | (0.114) |
| in house price prediction |  |  |  |  |
| N=1204; NT=6430 | First stage residual | 0.019*** | -0.353** | 0.076** |
|  |  | (0.003) | (0.156) | (0.034) |
|  |  |  |  |  |
|  |  |  |  |  |
| Panel (b) | Being a home owner | 0.159* | 1.260** | -0.543*** |
| Exclude income |  | (0.095) | (0.565) | (0.086) |
| N=1204; NT=6430 |  |  |  |  |
|  | First stage residual | 0.032 | -0.274 | 0.092*** |
|  |  | (0.032) | (0.191) | (0.026) |
|  |  |  |  |  |
|  |  |  |  |  |
| Panel (c) | Being a home owner | 0.095 | 1.931*** | -0.664*** |
| Head of Household |  | (0.102) | (0.604) | (0.147) |
| N=858; NT=4185 |  |  |  |  |
|  | First stage residual | -0.0220 | -0.560*** | 0.081** |
|  |  | (0.031) | (0.185) | (0.040) |
|  |  |  |  |  |
|  |  |  |  |  |
| Panel (d) | Being a home owner | 0.360** | 2.595*** | -0.704*** |
| Non-Head of Household |  | (0.153) | (0.934) | (0.178) |
| N=598; NT=2245 |  |  |  |  |
|  | First stage residual | -0.0120 | -0.806** | 0.122** |
|  |  | (0.0027) | (0.339) | (0.061) |
|  |  |  |  |  |
|  |  |  |  |  |
| Panel (e) | Being a home owner | 0.083*** | 2.955*** | -0.340* |
| Initial Employees |  | (0.015) | (0.939) | (0.179) |
| N=527; NT=2790 |  |  |  |  |
|  | First stage residual | -0.044*** | -1.096*** | 0.047 |
|  |  | (0.006) | (0.387) | (0.074) |
|  |  |  |  |  |
| Each panel contains a separate second stage model. Sample includes initial public renters only, 2000 – 2008. Number of observations (NT) and individuals (N) is shown in each panel. Bootstrapped standard errors, based on 2,000 replications, in parentheses. * p<0.10, ** p<0.05, *** p<0.01.  Column (3) reports marginal effects, from second stage models, calculated at the means of independent variables. The models contain all additional variables as reported in Table 3.  SAH=self-assessed health; GHQ=General Health Questionnaire; #Probs.=Number of self-reported health conditions. OLS=ordinary least squares; 2SRI = two-stage residual inclusion; Nbreg = negative binomial regression. | | | | |

| **Table B2**: Marginal Effects following Second Stage models of physical and psychological health outcomes, treating self-assessed health and ordinal and applying an ordered logit model | | | | | | |
| --- | --- | --- | --- | --- | --- | --- |
|  |  | (1) | (2) | (3) | (4) | (5) |
|  |  | SAH=1 | SAH=2 | SAH=3 | SAH=4 | SAH=5 |
| Panel (a): One stage model |  |  |  |  |  |  |
|  | Being a home owner | -0.0166*** | -0.0437*** | -0.0526*** | 0.0633*** | 0.0495*** |
|  |  | (0.0028) | (0.0072) | (0.0086) | (0.0104) | (0.0080) |
|  |  |  |  |  |  |  |
| Panel (b): Two stage model |  |  |  |  |  |  |
|  | Being a home owner | -0.0107* | -0.0283* | -0.0340* | 0.0409* | 0.0320* |
|  |  | (0.0058) | (0.0153) | (0.0183) | (0.0221) | (0.0173) |
|  |  |  |  |  |  |  |
|  |  |  |  |  |  |  |
|  | First stage residual | -0.0021*** | -0.0057*** | -0.0068*** | 0.0082*** | 0.0064*** |
|  |  | (0.0002) | (0.0005) | (0.0006) | (0.0008) | (0.0006) |
|  |  |  |  |  |  |  |
| Marginal effects calculated at the means of independent variables. The models contain all additional variables as reported in Table 3. Sample includes initial social renters only, 2000 – 2008. Bootstrapped standard errors, based on 2,000 replications, in parentheses. * p<0.10, ** p<0.05, *** p<0.01. | | | | | | |

| **Table B3:** Second Stage models of physical and psychological health outcomes for subsample of individuals with constant LSOA residence | | | |
| --- | --- | --- | --- |
|  | (1) | (2) | (3) |
| Outcome: | SAH | GHQ | #Probs |
| Model: | 2SRI OLS | 2SRI OLS | 2SRI Nbreg |
|  |  |  |  |
| Being a home owner | 0.155** | 2.243*** | -0.491*** |
|  | (0.066) | (0.725) | (0.126) |
|  |  |  |  |
| First stage residual | 0.048 | -0.477*** | 0.050* |
|  | (0.031) | (0.183) | (0.028) |
|  |  |  |  |
| Sample includes initial public renters (N=983; NT=3908) who keep the same LSOA of residence throughout their time in the sample only, 2000 – 2008. Bootstrapped standard errors, based on 2,000 replications, in parentheses. * p<0.10, ** p<0.05, *** p<0.01. In column (3), marginal effects, from second stage models, calculated at the means of independent variables are presented. The models contain all additional variables as reported in Table 3. | | | |

| **Table B4:** Linear model of whether baseline health predicts the decision to later become and owner | | | | |
| --- | --- | --- | --- | --- |
|  | (1) | (2) | (3) | (4) |
| Self-assessed health | -0.00108 | 0.00370 |  |  |
|  | (0.011) | (0.009) |  |  |
|  |  |  |  |  |
| GHQ Score | 0.00130 |  | 0.00138 |  |
|  | (0.002) |  | (0.002) |  |
|  |  |  |  |  |
| Number of health problems | -0.00202 |  |  | -0.00339 |
|  | (0.008) |  |  | (0.007) |
|  |  |  |  |  |
| Socioeconomic Characteristics | Yes | Yes | Yes | Yes |
| Year dummies | Yes | Yes | Yes | Yes |
| Locality dummies | Yes | Yes | Yes | Yes |
| Observations | 1204 | 1204 | 1204 | 1204 |
| Robust standard errors, clustered at local authority district level, included in parentheses. * p<0.10, ** p<0.05, *** p<0.01.  Sample is all individuals when first observed. Outcome variable =1 if individual i later goes on to own their house; =0 if always a renter.  Additional control variables are as reported in Table 3 (notes a and c). The results are also robust to including household characteristics.  The models are estimated using OLS for ease of interpretation. We have repeated using a logit model, and the resulting marginal effects are qualitatively similar to those presented above. | | | | |

**References for Appendix B**

Benítez-Silva H., Eren S., Heiland F., and Jiménez-Martín S. (2015) How well do individuals predict the selling prices of their homes? Journal of Housing Economics, 29: 12-25.

**Appendix C: How good are house price estimations**

The R-squared in the house price prediction model (Eq. 6) is just under 65%. In Table C1 we present selected coefficients to demonstrate that they behave as expected. For example, we can see that property value increases as the number of rooms within the property increases, and that all property types are less expensive than detached properties. Also, the predicted value increases with the council-tax band. These coefficients allow us to be confident in our house price imputation equation. (Full coefficients are available on request.)

In Figure C1 we plot the predicted house values (panel a) and the actual values reported by owners (panel b). The distributions of predicted and real values are similar, and this is confirmed in Figure C2 where we plot the predicted residuals. These residuals are normally distributed, with mean close to zero. The actual values display some clumping at £5,000 intervals.

In Figure C5 we present a scatter plot of land registry reported average house prices in LADs (x-axis) against our within-sample predicted averages (y-axis). If our predictions were perfect, we would expect all of the observations to lie on the 45-degree (grey, dashed) line. However, we can see that the actual relationship (black, solid line) is slightly above this, but that overall our approximations are quite good. The relationship is: average predicted values = constant + 1.07*(land registry averages), and the t-value on the 1.07 is 71.70, indicating it is strongly significant. However, we can also reject the hypothesis that the estimated coefficient is equal to one (t-value 4.94), implying whilst the relationship is close to unity, it is not equal to it.

Note Figure C3 is based on our estimation sample, whereas Figures C1 and C2 are based on owners only (i.e. out-of-sample). Figure C3 can be thought of as a test of whether predicted values of rented houses are the same as sold houses.

**Figure C1:** Predicted and reported house values


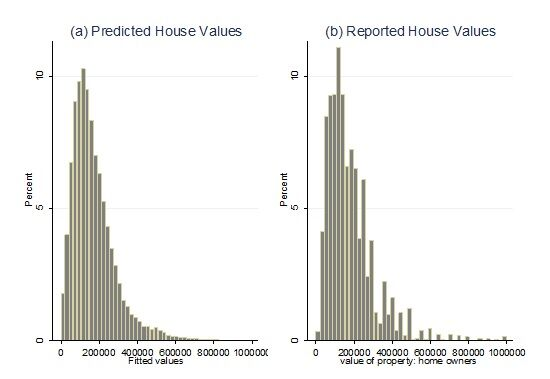


*Source: Authors' representation from BHPS data 1999-2008. For graphical clarity, we censor the observations at £1,000,000.*

**Figure C2:** Examining the goodness of predicted house values: predicted residuals


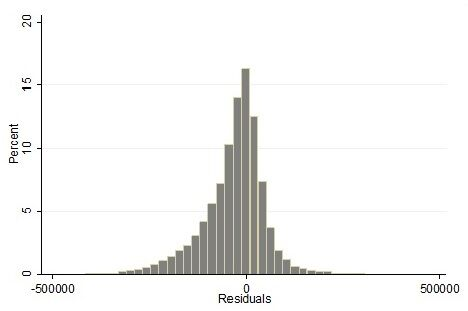


*Source: Authors' representation from BHPS data 1999-2008.*

**Figure C3:** Examining the goodness of predicted house values: local authority district average prices


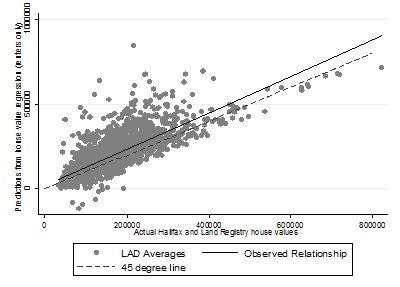


*Source: Authors' representation from BHPS data 1999-2008 (y-axis) and Halifax and Land Registry data (x-axis). For graphical clarity, we censor the observations at £1,000,000.*

| **Table C1**: Selected output from the hedonic house price equation | | |
| --- | --- | --- |
|  | Coefficient | Std. Error |
| Local average house prices | 0.94*** | 0.01 |
| Number of rooms: 1 | Reference category | |
| Number of rooms: 2 | 33682.62** | 14425.1 |
| Number of rooms: 3 | 86025.43*** | 15701.5 |
| Number of rooms: 4 | 127422.40*** | 17861.8 |
| Number of rooms: 5 | 171141.30*** | 20585.7 |
| Number of rooms: 6 | 224716.30*** | 23718.4 |
| Number of rooms: 7 | 287368.40*** | 27162.6 |
| Number of rooms: 8 | 343803.40*** | 30809.8 |
| Number of rooms: 9 | 392865.60*** | 34507.7 |
| Number of rooms: 10 | 635098.90*** | 38751.2 |
| Number of rooms: >10 ^a^ | Included |  |
| Type of accommodation: detached | Reference category. | |
| Type of accommodation: Semi-detached | -12676.18** | 5448.71 |
| Type of accommodation: End-Terrace | -16062.47** | 8968.31 |
| Type of accommodation: Terrace | -45912.85*** | 7109.76 |
| Type of accommodation: Purpose built flat | -58109.84*** | 16059.2 |
| Type of accommodation: Converted flat | -49361.92*** | 17839.6 |
| Council tax band: A | Reference category |  |
| Council tax band: B | 1895.92 | 2149.46 |
| Council tax band: C | 10447.62*** | 2199.59 |
| Council tax band: D | 20214.81*** | 2301.62 |
| Council tax band: E | 41516.69*** | 2715.82 |
| Council tax band: F | 73290.57*** | 3206 |
| Council tax band: G | 171740.90*** | 3471.27 |
| Council tax band: H | 283549.70*** | 6319.64 |
| Other household characteristics ^b^ | Yes | |
| Bootstrapped standard errors, clustered at local authority level and based on 2,000 replications, in parentheses.  * p<0.10, ** p<0.05, *** p<0.01.  a: We included the number of rooms up to 20. The coefficients are all monotonically increasing, and statistically significant at p<0.001.   b: Other controls include the central heating fuel type, if there is a separate toilet/bathroom, if the kitchen is open-plan, if there is a garden/terrace, if there is an indoor toilet, if there are neighbourhood problems with either crime/vandalism and/or pollution/the environment. We additionally interact the number of rooms with property type. The coefficient sizes and significance levels are available on request. We omit them here due to reasons of brevity. | | |
